# Clinical-Diagnostic and Therapeutic Advances in Feline Hypertrophic Cardiomyopathy

**DOI:** 10.3390/vetsci12030289

**Published:** 2025-03-19

**Authors:** Felipe Gaia de Sousa, Ana Cristina Ribeiro Mendes, Luisa Pimenta de Carvalho, Suzane Lilian Beier

**Affiliations:** 1 Department of Veterinary Clinic and Surgery, Veterinary School, Federal University of Minas Gerais—UFMG, Belo Horizonte 31620-295, Minas Gerais, Brazil; suzanelb@ufmg.br; 2 Department of Veterinary Medicine, Faculty of Veterinary Medicine, Pontifical Catholic University of Minas Gerais—PUC Minas, Belo Horizonte 30140-002, Minas Gerais, Brazil; anacrmen@pucminas.br (A.C.R.M.); luisa.carvalho.1404871@sga.pucminas.br (L.P.d.C.)

**Keywords:** cardiovascular, cats, heart failure, hypertrophy, cardiovascular pathophysiology

## Abstract

A comprehensive literature review was conducted to summarise existing evidence and the latest therapeutic advancements in feline HCM. The disease phenotype is characterised by the development of concentric hypertrophy, which is variable but often asymmetric, primarily affecting the left ventricle. The HCM is marked by diastolic dysfunction, resulting in a reduced intracavitary internal diameter due to the disordered alignment of cardiomyocytes and excessive collagen deposition, which thickens the myocardial tissue and impairs relaxation. There is strong evidence supporting the role of genetic mutations in sarcomeric genes in the development of HCM. Clinical signs vary but are frequently associated with congestive heart failure (CHF) and ATE. Diagnosis is based on a combination of clinical evaluation, laboratory tests, and imaging studies, although certain aspects of the disease remain insufficiently understood and require further research. Therapeutic management focuses on controlling clinical signs, slowing disease progression, and improving both quality of life and life expectancy. However, ongoing studies are essential to refine diagnostic strategies and explore novel treatment options for better disease management.

## Simple Summary

Feline cardiomyopathies are cardiovascular diseases that can lead to severe regional and systemic dysfunctions, with hypertrophic cardiomyopathy (HCM) being the most common phenotype. Genetic evidence has identified the involvement of mutations in the MYBPC3 gene, which encodes myosin-binding C protein. Among the affected breeds, the A31P mutation has been detected in Maine Coon cats, while the R820W mutation has been identified in Ragdoll cats. The clinical signs of HCM vary among affected cats and primarily involve cardiovascular and/or respiratory changes. Early diagnosis is essential and should include physical examinations, laboratory tests, and imaging assessments. Additionally, arterial thromboembolism (ATE) is a potential complication of HCM, and screening for its presence is recommended. Treatment strategies depend on the disease stage, with therapeutic intervention typically starting at stage B2. The management plan is tailored to each cat’s condition, aiming to control clinical signs, prevent disease progression, and improve overall quality of life.

## 1. Introduction

Currently, there has been a noticeable increase in the number of companion animals, particularly cats [1]. Consequently, changes in their lifestyle have led to a growing demand for specialised veterinary services [2]. Additionally, the rising life expectancy of pet cats may contribute to the increased prevalence of diseases [2]. Among these, cardiovascular diseases stand out due to their frequency and severity, warranting special attention [2]. Feline cardiomyopathies, particularly the hypertrophic phenotype, are recognised as one of the leading causes of mortality in cats [3,4,5]. The HCM is the most prevalent feline heart disease, with a well-established genetic basis, and is characterised by left ventricular hypertrophy with or without systemic complications [6,7,8,9].

Early diagnosis of feline HCM is crucial, as it allows for patient staging, ongoing monitoring, symptomatic management, and control of disease progression [10]. Some cats may remain asymptomatic, making recognition of the condition challenging for both owners and veterinary professionals [2]. Furthermore, underdiagnosis may lead to a lack of appropriate intervention and a consequent reduction in the patient’s life expectancy [11]. Various diagnostic methods are available to facilitate early detection and support clinical and therapeutic decision-making [5]. This article aims to provide an updated overview of feline HCM, including its characterisation, diagnostic approaches, and therapeutic management. It also reviews the 2020 guidelines established by the American College of Veterinary Internal Medicine (ACVIM) and examines new evidence that has emerged since the publication of the consensus.

## 2. Feline Hypertrophic Cardiomyopathy

Feline HCM phenotype is a condition characterised by hypertrophy of the cardiac musculature and diastolic dysfunction, primarily affecting the left ventricular (LV) chamber [5,12,13]. The severity of HCM can vary from mild to severe and may lead to significant haemodynamic consequences [5]. Initially, changes in muscle thickness are observed in the left ventricular region, particularly in the ventricular wall and papillary muscles, either partially or entirely [5]. As a result of cardiac hypertrophy, diastolic dysfunction develops, disrupting the heart’s normal function and leading to severe systemic complications. These complications include haemodynamic disturbances, excessive volume supply to tissues and organs, thrombus formation, and congestive conditions such as pulmonary oedema [4,14,15]. Echocardiographic examination is a valuable tool for assessing these abnormalities [16]. Among felines, HCM is the most common form of cardiomyopathy [17,18,19]. A study by Argenta et al. [20] reported a prevalence of 78%, making it the most frequently observed phenotype. The Cat Scan Study by Payne et al. [21] found an HCM prevalence of 14.7% (95% CI: 12.3–17.4%), whereas the Cat Scan II study [22] reported a higher prevalence of 37.4% (*n* = 40/107). Matos et al. [23] identified a prevalence of 28.4% (*n* = 29/102). HCM is associated with multiple cardiac and systemic alterations, including increased LV wall density, morphofunctional cardiac changes, and activation of compensatory mechanisms [4,5]. More recently, Seo et al. [24] reported a prevalence of 17.4%, with a greater emphasis on subclinical cases. However, data on the prevalence of HCM at different disease stages remain limited. In most studies, cats are classified based on the presence or absence of clinical signs. Various factors contribute to diagnostic challenges, including phenotypic overlap, difficulties in classification, and a high proportion of asymptomatic cases. Further research is needed to refine classification methods and improve disease detection.

**Figure 1:**
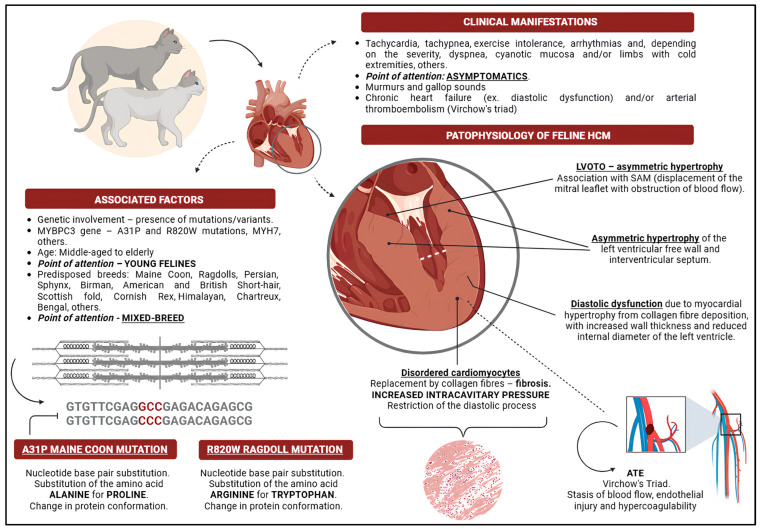
Schematic representation of the phenotype of feline HCM, including genetic, clinical, and pathophysiological factors associated with the development and progression of the disease. Consider: HCM—feline hypertrophic cardiomyopathy; A31P—A31P mutation; R820W—R820W mutation; LVOTO—left ventricular outflow tract obstruction; SAM—systolic anterior motion; ATE—arterial thromboembolism. BioRender 2025 (https://www.biorender.com/ (accessed on 10 January 2025)).

Feline HCM can affect cats of all breeds, but certain breeds are predisposed, including Maine Coon, Ragdoll, Persian, Sphynx, Birman, American and British Shorthair, Scottish Fold, Cornish Rex, Himalayan, Chartreux, and Bengal [3,25,26,27,28]. Some of these breeds have inherited mutations associated with HCM, such as the autosomal dominant mutation in Maine Coon and Ragdoll cats [4,6,7,29]. It is worth noting that purebred cats, such as Maine Coons and Ragdolls, may develop HCM due to genetic factors. However, mixed-breed cats can also be affected by the condition. Nonetheless, there is currently no documented evidence of mutations occurring in cats resulting from the blending of different breeds. According to Stern and Ueda [30], Maine Coon cats may develop echocardiographic abnormalities in the early stages of the disease (premature form of HCM). In some breeds, including Ragdoll and Sphynx cats, HCM can manifest aggressively and at a young age [31,32]. Age is another risk factor, with middle-aged and elderly cats being more susceptible, although cases have been reported across all age groups [3,21,22,26,27,28,33]. Matos and Payne [16] identified age over six years as an independent predictive factor for HCM. The condition is also more prevalent in males than females [19,23,34]. In their study, Matos and Payne [16] found that the mean diagnostic age was 7.3 years, with 23 out of 40 affected cats being male. Similarly, Seo et al. [24] reported a mean diagnostic age of 4.0 years (3.2–5.3), with a higher prevalence in male cats. Figure 1 provides a schematic overview of the phenotype of hypertrophic cardiomyopathy, including its clinical manifestations, associated factors, and pathophysiology.

## 3. Genetic Influence

Feline HCM has some similarities with human hypertrophic patterns [17,35]. According to Maron and Fox [17], “it is believed that no other animal species spontaneously develop HCM in such a closely aligned way with human HCM as cats”. Feline HCM was first identified in the mid-1970s [36], with genetic involvement being established in 1999 [33,37]. Mutagenic evidence suggests alterations in sarcomeric formation and composition [15], particularly in amino acid coding, which results from nitrogenous base pair substitutions [8,33]. These genetic modifications lead to changes in protein structure, expression, and function, ultimately contributing to disease development [8]. The first genetic mutation linked to feline HCM was identified by Meurs et al. [6] in a study involving 16 Maine Coon cats with a family history of HCM and a control group of 100 cats from various breeds.

A key genetic alteration in Maine Coon cats is the A31P mutation (A31P; p.A31P; G91C; c.91G > C), which involves a substitution of alanine for proline due to a single base pair change at codon 31 in exon 3 of the MYBPC3 gene [6,38]. This mutation has a reported prevalence of 22–42% in Maine Coon cats [33,39]. Stern et al. [38] found that most HCM-positive cats carried the A31P variant. While Meurs et al. [6] initially did not observe significant differences in disease severity between genotypes, Longeri et al. [25] and Ontiveros et al. [40] reported that homozygous cats exhibited more severe disease progression, with higher penetrance linked to age. Stern et al. [38] further supported these findings, showing that homozygous cats developed HCM more frequently than heterozygotes. Recent studies suggest that a loop-mediated isothermal amplification (LAMP) assay combined with a lateral flow dipstick (LFD) may serve as a rapid screening tool for detecting A31P mutations [41].

In 2007, Meurs et al. [7] identified a second variant in the MYBPC3 gene, R820W (c.2460C > T; R820W), which results in the substitution of arginine for tryptophan at codon 820 (p.R820W). This mutation affects sarcomeric structure and function. Kittleson et al. [33] reported that the R820W mutation occurs in 27% of Ragdoll cats and is exclusive to this breed [25]. Studies on disease severity have shown mixed results: Borgeat et al. [42] found that homozygous cats had a shorter life expectancy, whereas a later study by Borgeat et al. [32] in 2015 did not observe significant differences in disease occurrence between homozygous and heterozygous cats. Stern et al. [38] demonstrated that breeding a heterozygous male with a homozygous female resulted in offspring with more severe and earlier-onset HCM.

Additional genetic variants have been implicated in HCM, including mutations in the MYH7 and ALMS1 genes. Meurs et al. [43] described a mutation in ALMS1, a gene associated with Alström syndrome, in Sphynx cats. This mutation (g.92439157G > C) in exon 12 leads to a glycine-to-arginine substitution, altering protein structure and contributing to muscle disorders and fibrosis in HCM-affected cats. A study by Turba et al. [44] found that the g.92439157 C variant was highly propagated (frequency > 0.50) in 136 Italian Sphynx cats, revealing studies that verified the frequency of the variant in relation to the occurrence of HCM. Akiyama et al. [45] investigated the prevalence of MYBPC3 and ALMS1 variants in Japanese cats, identifying cases of HCM in individuals carrying MYBPC3-A31P and ALMS1 p.G3376R mutations across different breeds. However, a recent study by Seo et al. [46] found that while 70.9% of sampled cats carried the ALMS1 variant, it was not directly associated with HCM. Another mutation, MYBPC3-A74T, has been detected in Bengal cats, supporting the hypothesis that it contributes to HCM in homozygous individuals, although evidence for heterozygous carriers remains limited [47].

Sukumolanan and Petchdee [48] reported the prevalence of MYBPC3-A31P (16.33%) and MYBPC3-A74T (24.45%) in 49 Maine Coon cats, with disease severity increasing alongside epidemiological factors such as age, weight, heart rate, and isovolumetric relaxation time (IVRT) [20]. The authors suggested that HCM in Maine Coons may be multifactorial, influenced by both genetic predisposition and underlying health conditions [10]. A novel genetic association has been proposed in Maine Coon cats, linking HCM to the TNNT2 intronic variant c.95-108G > A [49]. Schipper et al. [49] conducted an international study on allele frequencies and concluded that this variant does not hold significant predictive value for breeding programmes. More recently, Grzeczka et al. [50] highlighted a major obstacle in feline HCM genetics: most affected cats are mixed-breed, making it challenging to identify disease-linked variants. Table 1 summarises key genetic evidence associated with the hypertrophic cardiomyopathy phenotype.

## 4. Pathophysiology of Feline Hypertrophic Cardiomyopathy

The LV diastolic dysfunction initially impairs blood ejection mechanisms, reducing the volume of circulating blood [5,51]. In healthy cats, the body can readily compensate for these haemodynamic changes [5]. However, in cats with HCM, degenerated cardiomyocytes are replaced by fibrous tissue due to reduced myocardial blood supply and hypertrophy. This process involves collagen deposition as a result of structural breakdown, ultimately leading to myocardial fibrosis [32]. As a compensatory response, hypertrophy develops alongside alterations in ventricular compliance, further exacerbating fibrosis and functional impairment [5].

**Figure 2:**
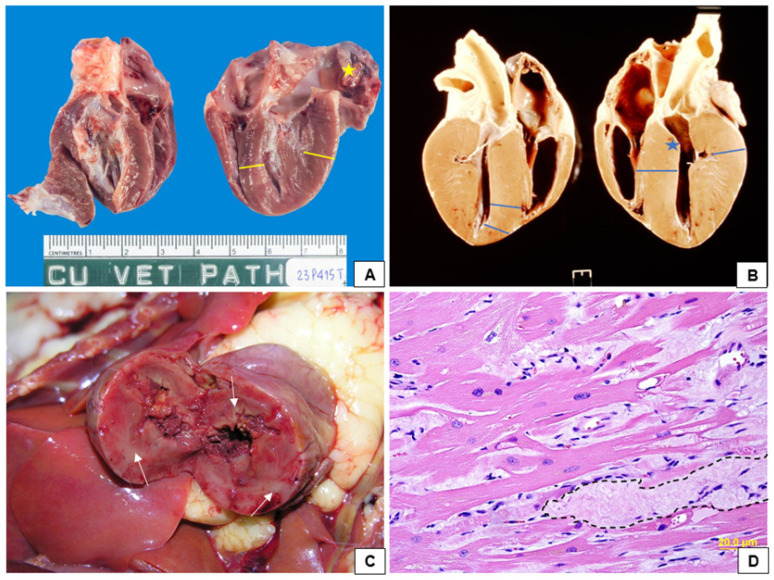
Macroscopic and histopathological changes in feline patients with hypertrophic cardiomyopathy. (**A**) The sectioned heart showing varying levels of hypertrophy of the left free ventricular wall and interventricular septum (yellow line); in the atrial region, it is possible to observe the presence of a thrombus (star). (**B**) Various levels of myocardial hypertrophy characteristic of heterogeneous hypertrophic conditions (blue lines) and septal hypertrophy at the level of the left ventricular outflow tract, which is a frequent finding in patients with outflow tract obstruction (star), were observed. (**C**) Cross-sectioned heart showing areas of cardiac fibrosis (arrows). (**D**) Histopathological evaluation of myocardial tissue showing muscle disarray and the presence of collagen fibre characteristic of a fibrotic process (dashed line). Images available in the Davis-Thompson Foundation database (Noah’s archive; (**A**). F64812, Heart: hypertrophic cardiomyopathy and thrombus of the left atrium, submitted by Sawang Kesdangsakonwut. Accessible at: https://davisthompsonfoundation.org/image-detail?image=F64812 (accessed on 10 January 2025). (**B**). F30288, Hypertrophic Cardiomyopathy, submitted by Williams. Accessible at: https://davisthompsonfoundation.org/image-detail?image=F30288 (accessed on 10 January 2025). (**C**). F32081, Hypertrophic Cardiomyopathy, submitted by Raquel Rech. Accessible at: https://davisthompsonfoundation.org/image-detail?image=F32081 (accessed on 10 January 2025). (**D**). F32760, Hypertrophic Cardiomyopathy (Histo), submitted by Raquel Rech. Accessible at: https://davisthompsonfoundation.org/image-detail?image=F32760 (accessed on 10 January 2025)).

Concentric hypertrophy reduces the ventricular lumen, prompting compensatory tachycardia in an attempt to maintain adequate blood pressure and cardiac output [30]. However, this increased heart rate shortens ventricular filling time, leading to further cardiac deterioration [30]. Severe regional and systemic complications, such as myocardial and organ ischaemia, may develop as a result [30]. Diastolic dysfunction can also lead to increased filling pressure as a compensatory mechanism, eventually reaching the threshold of maximum ventricular compliance [5]. Once this limit is exceeded, ventricular remodelling occurs due to sustained pressure elevation, which can secondarily affect the atria [5,16]. With disease progression, pulmonary capillary pressure may rise, leading to pulmonary oedema, pleural effusion, and impaired systolic function [52]. Additionally, right ventricular hypertrophy is a potential consequence of worsening clinical status and the presence of left-sided hypertension [53,54]. A common finding in cats with HCM is systolic anterior motion (SAM), in which the caudal portion of the mitral leaflet is displaced towards the LV outflow tract (LVOT), potentially resulting in LVOT obstruction (LVOTO) [16]. The SAM is often associated with mitral regurgitation due to altered valve coaptation, further complicating cardiac function [28]. Figure 2A–D illustrates the macroscopic and histopathological changes observed in hypertrophic cardiomyopathy.

According to Kittleson and Côté [5], mitral valve regurgitation and obstructive conditions in the LVOT can contribute to elevated atrioventricular pressure. In secondary cases of HCM, blood flow obstruction may arise due to several factors, including LVOT obstruction, displacement of the mitral leaflet, and increased pressure within the LVOT itself [55]. Cats with HCM often exhibit variable hypertrophic patterns, with some regions of the septum displaying greater hypertrophy than others. In many cases, LVOT obstruction results from interventricular hypertrophy [5,55]. Studies have demonstrated that hypertrophy tends to be more pronounced in the septal region adjacent to the mitral leaflets [56]. Emerging research has identified additional factors that may contribute to the pathogenesis of HCM, including fibrosis and myocardial remodelling. Joshua et al. [57] highlighted the involvement of specific signalling pathways, such as RhoGDI-RhoGTPase, integrin, LXR/RXR, PPARα/RXRα, HIF1α, and CXCR4, in myocardial transcription patterns associated with the disease.

This evidence suggests that variations in gene activation may play a role in HCM development. Colpitts et al. [58] observed notable differences in gene expression between young and adult cats, as well as between young and diseased HCM patients. Cats with HCM exhibited elevated levels of interleukins (IL-6) and monocyte chemoattractant protein 1 (MCP-1), along with increased IL-6 expression under thrombotic conditions. Age, lifestyle factors, and genetic transcription in adult cats may contribute to myocardial remodelling and thrombus formation, both of which are closely linked to HCM pathogenesis [58]. Rodríguez et al. [59] reported that cats with HCM exhibit reduced microvascular density, accompanied by increased interstitial oedema, collagen deposition, and mononuclear cell infiltration. The disease is also associated with an increased presence of resident macrophages, fibroblastic and/or vascular cells, and interstitial CD34+ cell groups. Given the established role of angiotensin II (ANG II) and angiotensin II-converting enzyme (ACE2) in cardiovascular disorders, Lean et al. [60] investigated ACE2 expression in both healthy and HCM-affected cats. The authors found that ACE2 was overexpressed in the cardiac tissue of affected cats, which may partially explain the regional and systemic consequences of HCM [60]. Increased ACE2 expression could potentially contribute to the progression of cardiovascular disease [60]. The increased expression of ACE2 may be capable of inducing cardiovascular disorders [60].

## 5. Clinical Manifestations

The clinical signs of HCM vary and are influenced by factors such as disease severity, age, and breed [4,5,17,22,26,55]. Affected cats may develop clinical signs ranging from CHF to ATE [4,17,22,26,30,54]. Some cats remain asymptomatic (15% in healthy cats, increasing to over 25% in those aged 9 years and older), making diagnosis challenging [24,35]. This diagnostic difficulty may stem from the low frequency of veterinary visits among owners and the absence of clinical signs in some cases [35]. Most clinical signs are related to cardiac and respiratory abnormalities, depending on the disease stage [61]. Common clinical signs include tachycardia, tachypnoea, exercise intolerance, and arrhythmias, with dyspnoea occurring in more severe cases [5,35]. The presence of a gallop heart sound may indicate underlying cardiac abnormalities but is not exclusive to HCM [28].

Cats with HCM and associated LVOTO have a higher risk of heart failure, ATE, and sudden death [3,27]. In the Cat Scan II study, the incidence of sudden death among cats with HCM was 5.9% [22]. Follby et al. [62] reported that HCM reduces life expectancy, with survival rates comparable to those of other non-cardiac diseases. Respiratory clinical signs, such as coughing, are less commonly observed, but pleural effusion can cause dyspnoea [63,64]. In cases of pulmonary oedema and pleural effusion [38], auscultation may reveal muffled heart sounds and crackles [9]. Systemic vascular congestion may lead to cyanotic mucosa due to reduced tissue perfusion [17,20,26,30,65].

Approximately 20–60% of affected cats develop heart murmurs of varying intensity, with prevalence increasing with age [21,24,27]. The cause of these murmurs may be mitral regurgitation or SAM of the mitral leaflet, which is commonly associated with LVOTO [5,35]. In a study by Payne et al. [21], 93.3% (42/45) of cats with SAM exhibited murmurs, and in other study [66] a third heart sound was detected in 40% (n = 20/50) of the evaluated animals. The study also found that older male cats with a larger body size and murmur grades of III/IV or higher were at greater risk of HCM. Compared with healthy cats (30–45%), murmurs were detected in approximately 80% of subclinical HCM cases, particularly in the parasternal region [21,27,67,68]. Pellegrino [69] noted that “not every cat with a murmur has cardiomyopathy, and the absence of a murmur does not rule out HCM”. Matos et al. [22] observed that murmurs were more frequent in cats with early-stage HCM, high ventricular thickness, reduced internal diastolic diameter, and lower shortening fraction (SF%) compared with healthy individuals. Tantitamtaworn et al. [70] suggested that assessing heart sounds and left atrium (LA) dimensions remains valuable in diagnosing HCM. If a cat is purring during auscultation, Vliegenthart and Szatmári [71] recommend placing a hand ventrally on the larynx to improve heart sound detection.

Due to diastolic dysfunction and reduced cardiac output, cats with HCM frequently develop ATE [4,72,73], a condition strongly associated with mortality [16]. Thromboembolic conditions arise from endothelial injury, activation of the coagulation cascade, and clot formation [74,75]. Shaverdian and Li [72], Poredos and Jezovnik [73], and Holm et al. [76] linked ATE to Virchow’s Triad, which consists of “blood stasis, a hypercoagulable state, and endothelial injury”. Thrombus formation is driven by blood flow turbulence, ejection disturbances, and vascular damage [75]. Thrombi most commonly develop in the aorta but can also be found in the renal, iliac, and femoral arteries [77,78]. The severity of vascular obstruction depends on the thrombus size, degree of blockage, and location [75,78]. Additionally, thrombi can trigger vasoconstriction, further reducing tissue perfusion [75,79]. According to Fox et al. [27], the incidence of ATE increases with age, peaking at 10 years before declining. The clinical signs of ATE vary depending on factors such as onset time, location, shape, size, and whether the obstruction is partial or complete [75]. Most affected cats exhibit hind limb paresis (unilateral or bilateral), reduced or absent femoral pulses, hypothermia, and cyanotic paw pads [75]. The ATE often results in ischaemic neuropathy due to vascular obstruction and is associated with significant pain [75,80,81].

As part of the differential diagnosis, symmetrical hypertrophy should be assessed to rule out angioendotheliomatosis, which can mimic HCM [82]. Other diseases, such as hyperthyroidism, may also cause cardiac changes that resemble the hypertrophic phenotype [24,83]. Measuring total thyroxine (T4) is recommended to differentiate hyperthyroidism from HCM. Lee et al. [84] described a Persian cat presenting with clinical signs of fatigue, tachycardia, and tachypnoea, initially diagnosed with hyperthyroidism. Echocardiographic evaluation revealed a hypertrophic phenotype, but hypertrophy regressed following hyperthyroidism treatment, demonstrating the potential overlap between these conditions [84]. Janus et al. [83] compared histopathological findings in cats with hyperthyroidism and HCM. They reported that hyperthyroid cats had an increased transverse heart diameter, whereas HCM-affected cats exhibited cardiomegaly, hypertrophy, and atrial appendage enlargement. Both groups demonstrated cardiomyocyte degeneration, but cardiac fibre disarray and more pronounced hypertrophy were exclusive to HCM cases [83]. Although hypertrophy induced by hyperthyroidism shares some similarities with HCM, it is typically less severe, aiding in the differential diagnosis.

## 6. HCM ACVIM Consensus

In 2020, the ACVIM proposed a classification system for feline heart disease [4], aiming to adapt the system developed by the European Society of Cardiology (ESC) [4,12]. However, the ESC classification has limitations, particularly as it does not account for cases where the phenotype changes throughout disease progression [12]. The ACVIM classification is based on structural, functional, or phenotypic characteristics rather than specific diseases [4]. This system ensures that cats are categorised according to observable structural and functional traits rather than being assigned a diagnosis based on a single disease process [4]. The key point of this classification is to ensure that animals are classified according to the present characteristics rather than a specific disease. It is also important to highlight that phenotypes overlap, meaning that a feline may initially present with an unclassified cardiomyopathy phenotype and, after some time, be reclassified as having a hypertrophic phenotype. The ACVIM consensus explicitly states that a **“**cat with left ventricular (LV) hypertrophy and hyperthyroidism is said to have an HCM phenotype in conjunction with hyperthyroidism” [4]. The classification system defines five cardiomyopathy phenotypes: HCM, restrictive cardiomyopathy (RCM), dilated cardiomyopathy (DCM), arrhythmogenic cardiomyopathy (ACM), and a non-specific phenotype that includes myocardial diseases that do not fit into the other categories [4].

Given its clinical relevance, the ACVIM system also incorporates a classification based on disease severity and the presence of clinical signs [4]. This system facilitates the monitoring and follow-up of affected cats at defined intervals, allowing veterinarians to determine the appropriate time to initiate treatment, evaluate therapy effectiveness, track disease progression, and establish prognosis [4]. The classification divides cats into five categories: A (predisposed cats), B (asymptomatic cats, further subdivided into B1 and B2), C (symptomatic cats, responsive to treatment), and D (cats with refractory disease, unresponsive to treatment) [4].

## 7. Diagnostic Tools for Feline HCM

### 7.1. Clinical-Laboratory Diagnosis

In addition to information obtained from anamnesis, including medical history, lifestyle factors, and observed clinical signs, a detailed clinical examination can provide valuable indications of HCM [5]. Cardiovascular abnormalities may subjectively suggest underlying pathology [9]. The presence of murmurs, abnormal breathing sounds, fatigue, tachypnoea, tachycardia, cyanotic mucosa, and cold extremities can all be clinical signs of cardiac disease [5,9,19,28]. Additionally, it is important to consider associated comorbidities, such as feline immunodeficiency virus (FIV) and feline leukaemia virus (FeLV), as they may influence disease progression [85]. Laboratory tests, including haematological and biochemical analyses, play an important role in assessing overall systemic health [86]. However, while these tests provide useful information, they are not pathognomonic for diagnosing HCM. Although speculation exists regarding the potential role of the neutrophil-to-lymphocyte ratio (NLR) in sudden death, Fries et al. [87] investigated its association with HCM and found that NLR levels were elevated in patients with CHF. However, the authors concluded that NLR cannot be considered a reliable prognostic marker for feline HCM. Certain conditions, such as hyperthyroidism and subaortic stenosis, can induce cardiac changes due to LV hypertrophy and should be ruled out during diagnosis [14]. Blood pressure measurements are also essential in differentiating HCM from other diseases, such as systemic hypertension and chronic kidney disease [4]. Unfortunately, despite its importance, blood pressure assessment is not yet a routine practice in all veterinary settings [88].

### 7.2. Imaging Diagnosis

#### 7.2.1. Thoracic Radiographs

Thoracic radiographs are considered a useful diagnostic tool for evaluating cardiovascular structure [54,89]. It is recommended that radiographic assessment includes two projections—laterolateral and ventrodorsal—to aid in diagnosis [90]. According to Guglielmini and Diana [90], chest radiographs provide a general characterisation of the condition, allowing for the assessment of the heart and other thoracic structures. Radiographic examination can help identify the consequences of HCM in other organs, including pulmonary oedema, pleural effusion, and bronchial compression [90,91]. Studies have shown that CHF caused by HCM more commonly leads to pulmonary oedema than pleural effusion [92]. However, diagnosing CHF via radiography has several limitations [93]. Rho et al. [94] reported that the accuracy of radiography in diagnosing HCM is approximately 70% and that automated assessment of residual architecture may improve diagnostic precision. Given the stress associated with handling and positioning, radiographs should only be performed once the patient is stabilised, ensuring that decompensated cats receive appropriate treatment before undergoing imaging [9,91].

Feline HCM is radiographically characterised by increased cardiac dimensions, particularly at the left atrioventricular level [90,95]. According to Pellegrino [69], “the results of chest radiography vary, depending on the observed hypertrophy, the degree of myocardial dysfunction, the presence of increased cardiac cavities and the severity of circulatory congestion”. Cardiac hypertrophy may be suspected through direct observation or by applying measurement strategies such as clock analogy and vertebral heart size (VHS) [90]. Radiographic findings may indicate cardiac alterations with or without complications [90]. Additionally, it may be possible to identify tortuous and dilated pulmonary vessels, which are potentially associated with elevated atrial pressure and increased pulmonary vascularisation [95].

Cardiomegaly with atrial enlargement is commonly observed in affected cats [95], although other conditions may also cause cardiac enlargement [90]. The “Valentine heart” sign, a radiographic indicator of cardiomegaly, is characterised by apical narrowing and broadening of the cardiac base [96,97]. The clock analogy method is based on the external contour of the heart, assigning chamber regions to corresponding positions on a clock face for systematic evaluation [90]. For mixed-breed cats, the normal VHS is 7.5 ± 0.3 vertebrae, with a maximum threshold of 8 [98], while for Maine Coons, it is 7.61 ± 0.34 [99]. Breed differences and thoracic conformation may lead to variations in mean VHS values [89]. In a study by Diana et al. [97], cats presenting with pulmonary oedema were found to have radiographic evidence of cardiomegaly, dilation of pulmonary vessels, particularly caudal vessels, and atrial enlargement, even in the absence of concomitant heart disease.

Kim et al. [93] conducted a study to assess the clinical impact of chest radiography in 78 cats (including 43 with HCM) and to identify predictive radiographic markers for HCM and CHF. They found that cats with HCM had a greater VHS (8.57 ± 0.74) compared to healthy cats, although no significant difference was noted in CHF incidence [93]. The sensitivity and specificity of VHS for CHF were reported as 76.19% and 40.91%, respectively, with positive and negative predictive values of 55.17% and 64.29% [93]. Radiographic signs such as LA enlargement and bulging of the left auricle significantly affected the left atrium-to-aorta (LA/Ao) ratio, which was found to be higher in affected cats, particularly those with CHF [93]. However, no significant difference was observed in vertebral left atrial size (VLAS) between CHF/HCM patients and healthy animals [93].

**Figure 3:**
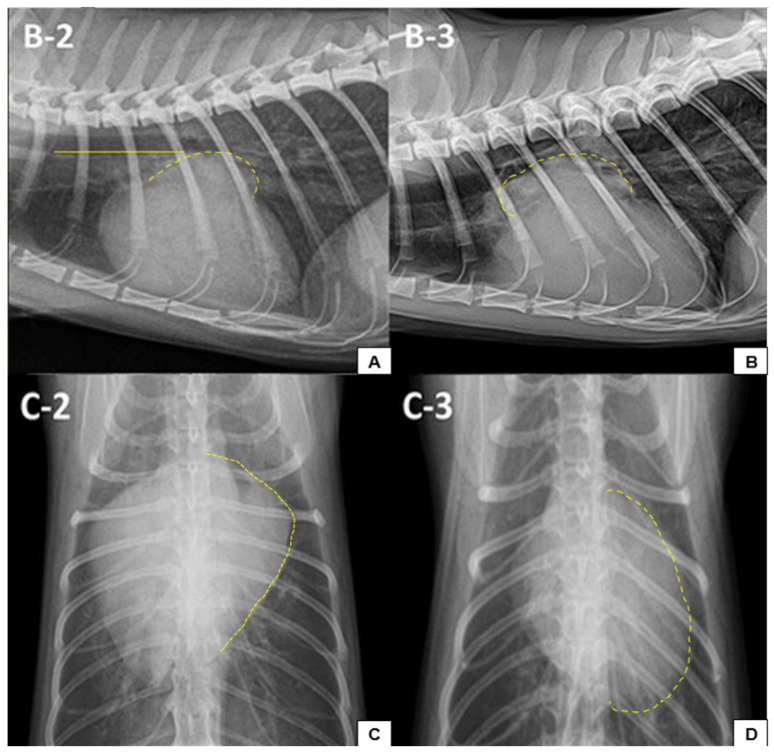
Radiographic images of cats with hypertrophic cardiomyopathy obtained in the study by Kim et al. [93]. (**A**,**B**) Lateral projection images and (**C**,**D**) ventrodorsal projection images. (**A**,**B**) Note the atrial enlargement (dashed line) and the parallelism of the trachea (line), showing tracheal displacement (**A**). (**C**,**D**) Atrial and ventricular enlargement with changes in the normal cardiac structure.

Cats with HCM frequently present with cardiomegaly, left atrial enlargement, and caudal pulmonary artery dilation, with elevation of the carina serving as a potential predictor of left atrial enlargement (94.12% specificity; 17.5% sensitivity) [93]. Kim et al. [93] also noted that dilation of the caudal pulmonary vein and left atrial enlargement differed between cats with CHF and those with HCM but without CHF. Their study highlighted a specific radiographic marker, stating that “the distal side of the summated shadow made by the right caudal PV with the ninth rib in HCM cats with CHF was significantly larger than that in HCM cats without CHF and a cut-off value of 5.35 mm was drawn with 75% sensitivity and 100% specificity”. These findings suggest that radiographic changes can provide valuable clinical and imaging insights for identifying cats with suspected HCM. Figure 3A–D illustrate the radiographic findings reported by Kim et al. [93].

#### 7.2.2. Electrocardiography

**Figure 4:**
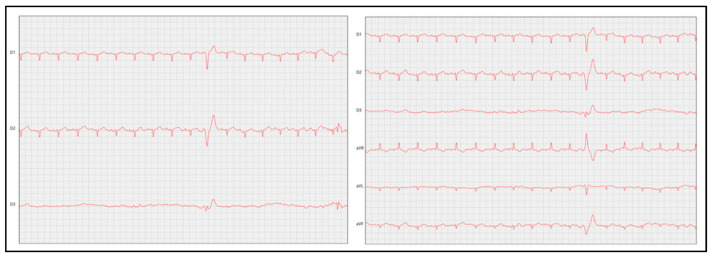
An electrocardiographic examination of a 10-year-old female mixed-breed feline patient diagnosed with stage B2 hypertrophic cardiomyopathy was performed. Note the presence of premature ventricular complex and right bundle branch blocks in the DII derivation.

Cats with HCM may exhibit electrocardiographic (ECG) abnormalities suggestive of the disease; however, these findings are not conclusive [55]. While ECG is a useful diagnostic tool, its sensitivity is limited, as different cardiac abnormalities can produce similar electrocardiographic patterns [55,95]. Cats with HCM may present with ventricular tachyarrhythmia, ventricular pre-excitation, wide QRS complexes, electrical conduction irregularities, increased R-wave amplitude, atrial fibrillation, and axis deviations, among other findings [3,5,9,100,101,102,103]. Additionally, some affected cats may develop supraventricular tachycardia, atrial flutter, atrioventricular block, bradyarrhythmias, or left anterior fascicular block (LAFB) [104]. According to Anderson [104] and Kaneshige et al. [105], LAFB is a common finding in cats with HCM and may be associated with myocardial fibrosis. More recently, Murphy and Nakamura [106] described cases of alternating ventricular pre-excitation with questionable pre-excitation complexes in cats with HCM. Repolarisation and depolarisation abnormalities remain rare in feline patients, particularly in relation to the hypertrophic phenotype [107]. The ST-segment elevation may occur but is typically transient [108]. Figure 4 illustrates the electrocardiographic findings of a feline patient with stage B2 hypertrophic cardiomyopathy.

A potential strategy for investigating ventricular arrhythmias in cats with HCM was explored by Bastos et al. [107], who studied the Tpeak–Tend (Tpte) interval, which measures the time between the peak and the end of the T wave, as well as the Tpte-to-QT interval ratio. The authors reported that the Tpte interval was longer in cats with HCM in leads II, aVR, aVL, and aVF and that a Tpte interval greater than 27.5 ms in lead aVF may help identify HCM with 83.3% accuracy. Cats with Tpte > 27.5 ms in leads aVR and aVF and >26.5 ms in lead aVL were more likely to have HCM, with odds ratios of 1.28, 12, and 1.16, respectively [107]. These findings suggest that evaluating the Tpte interval could assist in diagnosing left ventricular hypertrophy. Bastos et al. [109] further investigated the clinical utility of these indices, specifically in cats with HCM. Their study included 40 healthy cats and 23 affected cats, all of whom underwent 3–5 min of electrocardiographic monitoring [109]. Compared with healthy cats, affected cats exhibited short-term ventricular instability and prolonged QT and QRS intervals, suggesting that QT and corrected QT intervals, along with QRS complex duration, may serve as useful markers for HCM screening [109]. However, the potential for bradyarrhythmia following antiarrhythmic treatment remains an area requiring further research [110]. More recently, Cofaru et al. [111] emphasised the importance of electrocardiography in diagnosing HCM but highlighted that 24 h Holter monitoring would provide a more comprehensive assessment of electrical activity over an extended period [112]. Despite its advantages, Holter monitoring is not yet widely used in routine clinical practice.

#### 7.2.3. Echocardiography

**Figure 5:**
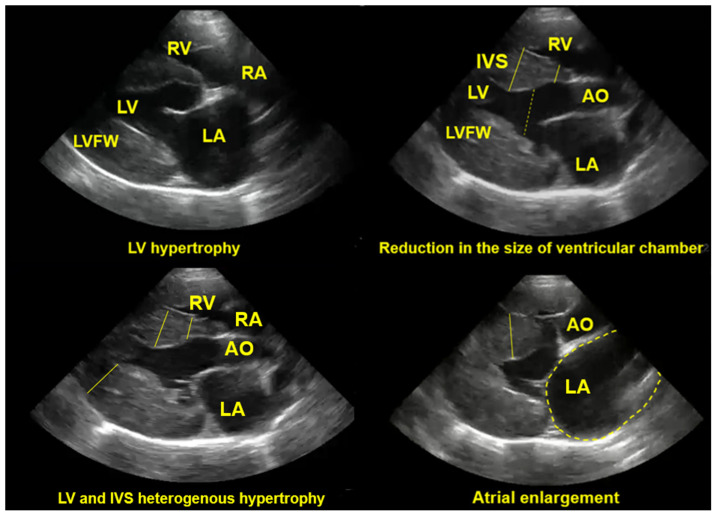
Echocardiographic examination showing right parasternal longitudinal 4 and 5 chambers in a feline patient diagnosed with hypertrophic cardiomyopathy. The images point to characteristics frequently observed in felines affected by the disease. There is evident left atrial dilation (dashed line), hypertrophy with a heterogeneous appearance in the region of the left ventricular free wall and interventricular septum (line), and a reduction in the size of the left ventricular cavity (dashed line). Consider: RA: right atrium; RV: right ventricle; LV: left ventricle; RV: right ventricle; AO: aorta; IVS: interventricular septum; LVFW: left ventricle free wall.

Considering that echocardiography is regarded as the gold standard for diagnosing HCM [4,5,10,35,70], it plays a crucial role in evaluating cardiac structures by assessing muscle hypertrophy (focal or regional) [28], particularly in the left ventricular region [16], and detecting mitral regurgitation associated with increased pressure [9,95]. Although echocardiographic examination is highly valuable, it requires experience and specialised training to ensure accurate interpretation [9,55,113]. Echocardiography can be performed using M-mode, 2D-mode, or a combination of both, though each technique has limitations [9,35,95]. Luis Fuentes et al. [4] and Wagner et al. [68] highlighted that challenges include heterogeneous imaging, such as focal hypertrophy and probe positioning artefacts, as well as non-interchangeable measurement values. To avoid false diagnoses and inaccuracies in measured parameters, careful attention must be given to the type of echocardiographic image obtained [28]. Figure 5 illustrates the echocardiographic alterations observed in a feline patient with hypertrophic cardiomyopathy.

The ACVIM consensus states that M-mode echocardiography remains the most commonly used method for LV measurement, particularly using short-axis imaging. However, this technique may lead to erroneous assessments, particularly in cases of heterogeneous hypertrophy [4,68]. Two-dimensional-mode echocardiography allows for the evaluation of LV musculature across different regions, capturing both short- and long-axis images to determine maximum myocardial thickness [4]. When using 2D mode, it is recommended to take measurements from at least two points in the septal region and ventricular wall while avoiding the papillary muscles [4,55]. Since measurements obtained from M mode and 2D mode cannot be directly compared, performing both methods is ideal to ensure accuracy [68]. To improve diagnostic reliability, it is recommended to calculate an average value from multiple echocardiographic measurements and assess myocardial motion changes [4,55]. Fries et al. [28] highlighted that ventricular hypertrophy alone should not be used as the sole criterion for diagnosing HCM, as certain cases—particularly those of secondary origin—may show reversible hypertrophy. Furthermore, Stern et al. [38] reported that homozygous cats more frequently exhibit preclinical signs of HCM than heterozygotes, including ventricular and/or septal wall thickening and an increased LA/Ao ratio. Cases of LVOTO are common in cats with HCM, particularly due to regional obstructions in the ventricular outflow tract, and are associated with increased blood pressure, particularly in wild-type genotype cats. However, Stern et al. [38] noted that the incidence of LVOTO was highest in cats with A31P HCM.

According to Luis Fuentes et al. [4], LV hypertrophy can be categorised as follows: <5 mm (normal LV), 5–6 mm (questionable), and >6 mm (hypertrophic LV). For left atrial (LA) evaluation, short-axis measurements can be obtained using the LA/Ao (left atrium/aorta) ratio in M mode and 2D mode at the end of ventricular systole/diastole [112,113,114,115], with variations reported between different genotypes [38]. Long-axis measurements are taken at the end of systole, extending from the interventricular septum to the LA free wall [116]. It is essential to assess the SF% of the LA, the blood flow velocity of the left atrial appendage (LAA), and the presence of spontaneous contrast or thrombi [18,91,117]. A study reported the following LA measurements at the end of ventricular systole: normal LA/Ao (<1.5), LA/Ao as a risk factor (>1.8), maximum LA diameter (16 mm), LAA velocity indicating blood stasis (<0.25 m/s), and LA SF% as a risk factor (<12%) [35]. In 2023, Matos et al. [118] reported that cats with advanced-stage HCM may exhibit thin and hypokinetic myocardial segments. While the ACVIM consensus does not specify standard echocardiographic measurement values, Luis Fuentes et al. [4] outlined echocardiographic protocols for assessing cats suspected of having HCM. Fries et al. [28] recommended performing Doppler echocardiography to evaluate left ventricular hypertrophy, SAM, LVOTO, diastolic dysfunction, atrial function and structure, and the presence of spontaneous contrast (“smoke”) and thrombi. Additionally, spectral and tissue Doppler imaging should be used to assess diastolic function, with tissue Doppler being the preferred method [28]. Recently, Colakoglu et al. [119] investigated the relationship between spontaneous contrast (“smoke”) and LA dimensions. Their findings suggest that in cats with HCM and smoke, there was a reduction in LA SF% and fractional area, both of which may serve as predictive markers for ATE.

In 2015, Payne et al. [21] conducted the Cat Scan study, screening 780 apparently healthy cats to identify predictive factors for HCM using echocardiography. Of these, 115 cats were diagnosed with HCM, with only 5.8% exhibiting SAM. However, among the 45 cats with SAM, 41 (91.1%) had HCM, suggesting a strong association between SAM and the disease. Cats with SAM and HCM had higher median values for left ventricular myocardial wall thickness, shortening fraction, and atrial diameter compared to those without SAM. Specifically, in HCM-positive cats with SAM, the median values for end-diastolic ventricular diameter and SF% were greater, although left atrial diameter and LA/Ao ratio remained unchanged. The study also found significant associations between HCM and factors such as age, increased blood pressure, male sex, weight, and body mass. Seo et al. [120] later reported that the presence of baseline SAM was more strongly associated with disease progression than its absence.

In 2022, Matos et al. [22] conducted Cat Scan II, a multicentre study using data from 107 cats from leading veterinary centres across Europe. Over an average follow-up period of 5.6 years, the authors observed no changes in ventricular wall thickness but did report increases in atrial size and diastolic ventricular internal diameter [22]. The prevalence of SAM remained stable, with 33% of cats presenting SAM at the first assessment and 29% at the second assessment. Additionally, the study noted the presence of focal ventricular wall thinning, atrial dilation, hypokinesia, and asymmetric hypertrophy [22]. Key predictive variables identified in Cat Scan II included a reduction in atrial ejection fraction (<25%), an increase in ventricular ejection fraction, and a decrease in internal diastolic ventricular volume in cats with HCM. Notably, cats with an atrial ejection fraction above 25% had a 91% lower probability of developing HCM [22]. Based on these findings, the authors concluded that a low initial atrial ejection fraction (<25%), an increased ventricular fraction, and high body weight were independently associated with the development of HCM [22].

In addition to the standard diagnostic measures for feline HCM, Seo et al. [121] conducted a study to evaluate the dimensions of the mitral valve leaflets and their potential association with disease development. Their findings suggested that anterior mitral valve elongation may serve as a predictive marker for HCM [118]. However, a study by Velzen et al. [122] found that mitral leaflet size was not a significant predictor of myocardial hypertrophy. Other studies have identified decreases in mitral and tricuspid annular plane systolic excursion values [123], hypokinesia, and echocardiographic contrast (“smoke”) as potential indicators of worse clinical outcomes in HCM [92,118]. However, there remains a lack of studies investigating whether diastolic dysfunction can be considered a predictive factor for HCM [124].

Aortic annular plane systolic excursion is frequently reduced in cats with HCM, making it a diagnostic marker of interest [125]. Bach et al. [126] emphasised the importance of assessing mitral annular displacement using Doppler tissue tracking (TT-LD) and M-mode techniques to evaluate CHF. Their study reported a reduction in longitudinal displacement measured by M-mode-derived mitral annular plane systolic excursion (MAPSE) and TT-LD, correlating with the presence of CHF [126]. While TT-LD was found to be more sensitive than MAPSE, the authors noted that the two methods are strongly correlated but not interchangeable [124]. In a study by Matos et al. [23], septal thickening was evaluated in non-referred cats, revealing that elderly cats exhibited greater myocardial free wall thickness than younger cats. Approximately 38% of the cats (n = 11/29) presented with SAM, with an average myocardial thickness of 6.5 mm. Most cats had normal LA/Ao ratios and atrial dimensions, with an SF% of 24%. The aortoseptal angle in cats with HCM was 140 ± 14.4°, suggesting that aortoseptal angulation assessment may be a useful parameter to include in echocardiographic evaluations of HCM in cats [127].

Another diagnostic approach in echocardiographic evaluation is strain imaging, a technique used to analyse cardiac deformation by mapping myocardial movement and displaying graphical representations of changes during the cardiac cycle [28]. Spalla et al. [128] conducted a study using strain imaging in cats with HCM, reporting that subclinical HCM cases exhibited abnormalities in both the long and axial axes. These findings highlight the potential role of strain imaging as an additional diagnostic tool for detecting early myocardial changes in affected cats.

Saito et al. [129] conducted a study to evaluate myocardial function in cats with HCM and with and without LVOTO using speckle-tracking echocardiography. Their findings showed that cats with HCM exhibited reduced longitudinal (epicardial and endocardial) and circumferential (epicardial) left ventricular deformation, although no significant differences were observed between patients with and without LVOTO [129]. However, in HCM cats with LVOTO, the endocardial and entire circumferential myocardial regions displayed reduced deformation, suggesting that left ventricular myocardial function was more impaired in these cases [129]. More recently, Hirose et al. [130] reported that LVOTO leads to changes in the intraventricular pressure gradient. However, this parameter is calculated using Euler’s formula rather than conventional echocardiography, which may serve as an alternative method for evaluating cardiac function.

Suzuki et al. [131] investigated the relationship between CHF and myocardial function using speckle tracking in both healthy and diseased cats. Their study found that cats with CHF exhibited an increased LA/Ao ratio and a reduced left ventricular apical circumference. Additionally, CHF patients showed an increase in left ventricular internal diameter at the end of diastole and a decrease in longitudinal strain of the right ventricle. Left atrial dilation and reduced left ventricular apical myocardial function were identified as potential predictive markers for CHF. Furthermore, their findings suggest that left ventricular dilation and dysfunction may contribute to the development of CHF in cats with asymptomatic disease.

### 7.3. Biomarkers and Other Diagnostic Options

Biomarkers are substances that can be measured through blood exams to assess cardiac function and damage [20,28,132]. They aid in characterising disease progression and evaluating disease severity and can serve as indicators of prognosis [32,86,132]. However, their availability, cost, and the time required for results may limit their widespread use [4,5,32,35]. The primary biomarkers used in cardiac evaluation include N-terminal pro-atrial natriuretic peptide (NT-proANP), atrial natriuretic peptide (ANP), N-terminal pro-B-type natriuretic peptide (NT-proBNP), B-type natriuretic peptide (BNP), and cardiac troponin-I (cTnI) [9,32,35,55,86,133]. These biomarkers can be categorised into injury markers (cTnI) and functional markers (NT-proANP and NT-proBNP). NT-proANP and NT-proBNP are released into the bloodstream in response to cardiac overload and/or distension [32,86]. Heishima et al. [134] studied ANP levels in control cats (n = 78) and cats with cardiomyopathy (n = 83) and found that ANP concentrations increased as the disease progressed. However, the authors cautioned that these data should not be used in isolation for diagnosis [134].

NT-proBNP, the most widely used cardiac biomarker [28], is particularly useful for differentiating causes of dyspnoea due to its long half-life and stability [10,61,92,135]. It is commercially available for felines through the Feline SNAP test. According to Kittleson and Côté [5] and Lu et al. [136], this test is not recommended as a screening tool for healthy cats. However, Luis Fuentes and Wilkie [35] suggest that it is valid for cats at high risk of HCM. Fries et al. [28] emphasised that NT-proBNP is not exclusive to cardiac diseases, and further diagnostic tests, such as echocardiography or magnetic resonance imaging, are necessary for a definitive diagnosis. Multiple studies have demonstrated elevated NT-proBNP and NT-proANP levels in cats with HCM [133,137,138,139], with higher NT-proBNP levels increasing the risk of cardiac events [138]. In Matos et al. [22], the average NT-proBNP concentration was 400 pmol/L, while Stern et al. [38] found that NT-proBNP levels were associated with the A31P mutation, LVOTO, and HCM diagnosis, with higher concentrations observed in homozygous cats.

For assessing cardiac injury, cTnI levels are used to detect myocardial damage [10], and its measurement is particularly beneficial in HCM cases [28]. Additionally, cTnI is considered a prognostic marker, as higher concentrations correlate with shorter survival times [42,140]. Gavazza et al. [85] reported that cTnI levels increase with cardiac disease severity, although they do not definitively indicate the underlying cause of myocardial injury. Hori et al. [140] found that cTnI concentrations can reflect the severity of heart disease, provided that other potential causes are excluded. Hanås et al. [141] observed that cTnI levels in HCM patients were positively associated with left ventricular free wall thickness and the LA/Ao ratio, with higher concentrations seen in cats with atrial enlargement. However, Stern et al. [38] found that cTnI levels did not differ between genotypes. Seo et al. [142] reported that NT-proBNP and cTnI levels were significantly elevated in cases of SAM, further supporting their use as biomarkers for disease progression.

Additional biomarkers may provide further diagnostic insights. Endothelin concentration measurements can be useful, as endothelins are potent vasoconstrictive peptides produced in vascular endothelial cells, but their clinical significance remains moderate [143]. For cases of ATE, D-dimer is a useful biomarker for assessing coagulation function, as cats with HCM exhibit elevated levels [144]. Additionally, proteomic analysis has revealed the upregulation of integrin alpha M subunit (ITGAM), elongin B (ELOB), and fibrillin 2 (FBN2) in HCM, suggesting that these molecular markers may aid in further understanding disease pathophysiology [144].

Genetic testing can be performed to identify mutations associated with HCM [6,8]; however, these tests must be conducted on an individual basis for each patient [28]. Deoxyribonucleic acid (DNA) analysis and genetic sequencing can be carried out using blood samples or oral mucosal swabs [6,145]. Importantly, due to the variety of mutations observed [8], a negative result does not exclude the possibility of HCM [145]. Genetic mapping of predisposed breeds would be valuable, particularly given that studies indicate 39.4% of Maine Coon cats and 27% of Ragdolls carry MYBPC3 gene mutations [146]. As a result, periodic monitoring of these breeds is recommended [28].

Emerging evidence suggests that remodelling mediators such as lumican, lysyl oxidase (LOX) isoenzymes and isoforms, and transforming growth factor beta (TGF-β) play roles in hypertrophy and fibrosis [145]. Research has shown that collagen and non-collagen components, as well as mononuclear cell infiltration, are significantly increased in HCM-positive cats [147]. These cats exhibit elevated concentrations of lumican, LOX, TGF-β1, and TGF-β2, indicating a link between remodelling and fibrosis markers and HCM pathophysiology [147]. The authors suggest that measuring these substances could be a useful strategy in future studies to improve disease characterisation and aid in developing potential treatment strategies. Additionally, Kaplan et al. [148] highlighted that, in some cases, fibrosis becomes self-perpetuating, independent of myocyte dysfunction, and may be driven by pleiotropic events, leading to further increases in fibrosis markers.

A new potential biomarker for cardiac fibrosis, galectin-3, a beta-galactoside-binding lectin derived from macrophages, has been identified. Stack et al. [149] conducted a study involving 80 cats, of which 56 were in ACVIM B1 and C stages. Their findings showed that galectin-3 levels were significantly higher in cats with HCM compared to healthy controls, with a strong correlation between galectin-3 concentration, left atrial dimensions, LA/Ao ratio, and extracellular volume [147]. Fries et al. [150] further reported that HCM-positive cats have increased extracellular volume, signs of diastolic impairment, and larger left atrial dimensions. Deemekul et al. [151] found that galectin-3 is associated with changes in the interventricular septum and shortening fraction values, suggesting its potential as an early predictive biomarker for HCM. However, while these findings support its diagnostic value, further research is needed to clarify its role in disease progression. Recent studies have also identified myocardial gene biomarkers, including interleukin 18 (IL-18), insulin-like growth factor binding protein 2 (IGFBP-2), and WNT Family Member 5A (WNT5A), which appear to be elevated in both clinical and subclinical HCM cases [152].

Because HCM increases the risk of ATE due to blood stasis, Li et al. [153] investigated the potential role of neutrophil extracellular traps (NETs) as biomarkers by measuring cell-free DNA (cfDNA) and citrullinated histone H3 (citH3). Their findings indicated that cats with HCM and ATE exhibited increased cfDNA levels, with citH3 detected in 52% of affected animals, though at lower concentrations in those with ATE. Li et al. [153,154] concluded that NETs may serve as a potential diagnostic tool for detecting thrombi in HCM-positive cats, though variability in thrombus distribution—influenced by factors such as shear stress and neutrophil activation—remains a consideration. Recently, Shaverdian et al. [73] introduced a novel flow cytometry technique utilising procoagulant markers such as thrombin and convulxin, which demonstrated an increased procoagulant tendency in cats with HCM. These findings suggest that further investigation into coagulation markers and NETs may provide valuable insights into the thrombotic risks associated with feline HCM.

## 8. Treatment Strategies

In general, cats with HCM phenotype should be treated according to disease stage [4,5]. Given the prevalence and mortality rates, it is crucial that diagnostic and therapeutic management be effective [28]. The prognosis of HCM varies depending on disease progression [28]. One of the key objectives of therapy is to identify cats at higher risk of developing HCM, particularly those with comorbidities or an imminent risk of sudden death [16,148]. Additionally, minimising stress is a priority in treatment, as stress can exacerbate the disease [155]. Cats often experience stress during laboratory tests, physical examinations, and imaging procedures, and oral medication administration can further aggravate their condition [155]. Stress control is essential to prevent clinical deterioration or decompensation [91]. Therefore, cat-friendly veterinary practices should be implemented from the moment the cat arrives for a routine appointment [155].

The ACVIM consensus recommends different therapeutic strategies depending on the disease stage, although many of these strategies have limited scientific evidence [4]. Fries et al. [28] emphasised that prognosis and treatment options should be individualised based on each patient’s specific disease characteristics. Stage A cats (predisposed but asymptomatic) do not require treatment [4]. Stage B1 cats (asymptomatic with mild changes) also do not require treatment, but annual monitoring is recommended to assess disease progression [4,5]. For Stage B2 cats (asymptomatic with moderate to severe heart changes), thromboprophylaxis and regular monitoring are advised [53,72,156]. Thromboprophylaxis is recommended when there is a high risk of ATE, particularly in cases of moderate to severe left atrial dilation, reduced SF%, and decreased left atrial appendage velocities [4,95,156]. In these cases, clopidogrel (18.75 mg/cat orally every 24 h with food) is recommended, either alone or in combination with aspirin (25 mg/kg PO every 48–72 h; 81 mg PO every 72 h; or 5 mg/kg PO every 48 h) or factor Xa inhibitors such as rivaroxaban (2.5–5 mg PO every 24 h) [4,72]. Recently, Kamp et al. [157] reported a case of hepatic complications associated with clopidogrel, which, although rare, warrants further investigation. For ventricular arrhythmias or atrial fibrillation, the use of atenolol (6.25 mg/cat PO every 12 h) or sotalol (10–20 mg/cat PO every 12 h) is recommended [4,158]. In a study by Kortas and Szatmári [159] that evaluated atenolol use in 23 cats, 47% showed a positive response in reducing SAM; however, only 9% maintained a long-term response when administered every 12 h. Approximately 26% of atenolol-treated cats died from cardiac-related causes, but it remains unclear whether non-responsive cats had HCM or congenital heart defects [159]. The use of pimobendan, angiotensin-converting enzyme (ACE) inhibitors, and spironolactone is not recommended [4,160,161]. Regular heart rate monitoring is advised [4].

For Stage C cats (symptomatic with CHF), immediate management should focus on oxygen supplementation and stress minimisation. If required, butorphanol (0.2 mg/kg IV) can be administered for sedation [4,5,9]. Additional treatments include furosemide (continuous rate infusion [CRI] at 0.6–1 mg/kg/h or bolus at 1–2 mg/kg), even in cases of azotaemia [4,90], thoracentesis in cases of pleural effusion [91], and pimobendan (0.625–1.25 mg/cat PO) in cases where there are signs of low cardiac output, cold extremities, and absence of LVOTO [4,91]. If pimobendan is ineffective, dobutamine (5–15 μg/kg/min IV) may be used [4]. Atenolol, transdermal nitroglycerin, and ACE inhibitors are not recommended for Stage C cats [4,91]. These patients should be discharged as soon as possible to reduce hospital-associated stress [91]. Renal function and respiratory rate should be reassessed 3–7 days post-discharge, with an at-home target respiratory rate of <30 breaths per minute [4].

For Stage C cats with chronic CHF, furosemide is recommended at doses of 0.5–2 mg/kg PO every 12 h, adjusting the dose to maintain a resting respiratory rate below 30 breaths per minute. Regular monitoring, including urea, creatinine, and electrolyte levels, should be performed 3–7 days after initiating treatment. In cats with respiratory distress, the intravenous administration of furosemide is advised [4]. Thromboprophylaxis with clopidogrel (18.75 mg/cat PO every 24 h with food) is also recommended [79]. Pimobendan can be administered at 0.625–1.25 mg/cat PO every 12 h if systolic dysfunction is present and there is no evidence of LVOTO [162]. Routine monitoring, including at-home respiratory rate measurements, is essential while minimising patient stress during follow-ups [4]. Some studies have suggested there are potential arrhythmic effects of oral pimobendan in healthy cats, raising concerns about whether intravenous administration may increase this risk. However, research to date indicates that pimobendan does not appear to influence arrhythmia development, though further investigation is needed [163].

Regarding pimobendan use in HCM, Oldach et al. [164] conducted a study to evaluate its pharmacological effects and tolerance in cats with HCM. The study assessed 21 cats before and 90 min after receiving a single dose of 1.25 mg of pimobendan, alongside 7 control cats. The results showed that pimobendan acutely increased left atrial and ventricular systolic function, as well as LVOT velocity, without causing adverse effects [164]. However, its benefits for left atrial function remain uncertain and require further study [165]. Schober et al. [166] investigated the effects of pimobendan in cats with CHF and found that approximately 32% of cats with non-obstructive HCM and 28.6% of cats with obstructive HCM showed clinical improvement. However, the study concluded that pimobendan use within 180 days did not provide significant benefits for cats with HCM and CHF, suggesting that its effectiveness in obstructive and non-obstructive HCM should be further evaluated [164].

For Stage D cats, the following treatments are recommended: torasemide (0.1–0.2 mg/kg PO every 24 h) in cases where furosemide doses exceed 6 mg/kg/day PO [4]; spironolactone (1–2 mg/kg PO every 12–24 h) [167]; and pimobendan (0.625–1.25 mg/cat PO every 12 h) for global left ventricular dysfunction [168]. Torasemide, when administered as a single dose in cats, has been shown to enhance diuresis and the function of the renin–angiotensin system (RAS), demonstrating high bioavailability with minimal adverse effects [169]. Additionally, dietary supplementation with taurine (250 mg PO every 12 h) is recommended [170]. High-sodium diets should be avoided, body weight should be monitored, and cachectic patients should be closely managed [91]. Continuous monitoring of electrolyte levels is crucial, as potassium supplementation may be required if levels decline [4,91]. Recent studies suggest that the acylcarnitine-to-free carnitine ratio is lower in cats with HCM, indicating potential myocardial metabolic alterations in the early stages of the disease [171]. However, further investigations are needed to validate these findings.

In cases of ATE, the possibility of euthanasia should be carefully evaluated due to the severity of the condition and potential poor prognosis [42]. Pain management is a priority and can be achieved using opioids such as fentanyl (2.5 μg/kg bolus|4–10 μg/kg/h CRI), hydromorphone (0.08–0.3 mg/kg subcutaneously [SQ], intramuscularly [IM], or intravenously [IV] every 2–6 h), and methadone (0.1–0.3 mg/kg IV/IM every 12 h) [4,9]. Anticoagulant therapy includes low-molecular-weight heparin (100–200 IU/kg SQ every 12–24 h or 1.0–1.5 mg/kg SQ every 12–24 h), unfractionated heparin (150–250 IU/kg SQ every 6–8 h; 250 U/kg SQ every 6 h), or rivaroxaban (2.5–5 mg PO every 24 h), a factor Xa inhibitor [72,75]. Heparin may be substituted with a factor Xa inhibitor combined with clopidogrel [4]. However, thrombolytic therapy is not recommended in cats with ATE due to the high risk of complications and limited efficacy [172,173,174,175].

Lo et al. [175] conducted a study assessing the adverse effects of combined clopidogrel (18.75 mg PO every 24 h) and rivaroxaban (2.5 mg PO every 24 h) in 32 cats with ATE, intracardiac thrombosis, or spontaneous echocardiographic contrast (“smoke”). Among the treated cats, five exhibited mild adverse effects, including epistaxis, haematemesis, haematochezia, or haematuria, but no severe complications were reported [175]. The median survival time was 257 days for all cats, 502 days for those with ATE affecting one or more limbs, and 301 days for cats with ATE in a single limb [175]. The ATE recurrence rate was 16.7% during treatment, and no cats developed new thrombi when treated with the dual therapy, demonstrating the potential benefits of the combination therapy [175]. In a subsequent study, Lo et al. [176] evaluated the effects of clopidogrel, rivaroxaban, or a combination of both in nine cats and found that the dual therapy more effectively reduced platelet activity, agonist response, and thrombin production compared to either drug alone. More recently, Jaturanratsamee et al. [177] reported that treatment with rivaroxaban (2.5 mg/kg PO every 24 h) or a combination of enoxaparin (1 mg/kg injectable) with clopidogrel (3 mg/kg PO every 24 h) for two months prevented thrombus formation in cats with HCM. Emerging therapies, such as platelet integrin αIIbβ3 antagonists (abciximab and eptifibatide) and NET inhibitors, show promise for thromboprophylaxis, though further research is required [72].

Clopidogrel is recommended as a first-line treatment for ATE and should be initiated at an initial oral dose of 75 mg, followed by a maintenance dose of 18.75 mg PO every 24 h [4,68]. According to Matos and Payne [16], clopidogrel is the preferred medication for preventing ATE recurrence. However, Rishniw [178] noted that clopidogrel reduces ATE risk by only 3–4% in cats with moderately severe HCM, raising questions about whether the benefits outweigh the challenges of administration in feline patients. In cats with ATE and concurrent CHF, treatment includes furosemide (1–2 mg/kg IV bolus|0.6–1 mg/kg/h CRI) and oxygen supplementation as needed [91]. Follow-up reassessments are conducted 3–7 days post-discharge and again 1–2 weeks after the ATE event [4]. Due to stress susceptibility in feline patients, monitoring intervals typically range from 1 to 3 months, balancing clinical oversight with stress reduction [4].

New therapeutic strategies have been developed to promote myocardial relaxation, reduce obstructive conditions, and mitigate fibrotic events in HCM [148]. Among these, two small-molecule inhibitors, mavacamten and aficamten, have been studied for their effects on myosin ATPase, which releases energy for muscle contraction [148]. Studies in mouse models have shown that mavacamten prevents ventricular hypertrophy, cardiomyocyte disorganisation, and fibrosis. When administered to mice with HCM, it was found to reduce ventricular wall thickness [148]. Unlike beta-blockers and calcium channel blockers, mavacamten decreases the percentage of fractional shortening (FS%) without affecting heart rate [148]. Aficamten, on the other hand, has demonstrated a reduction in pressure gradients and IVRT, contributing to beneficial diastolic effects [148].

In a study by Sharpe et al. [179], five mixed-breed Maine Coon cats were treated with 2 mg/kg aficamten or placebo, and echocardiographic assessments were performed 48 h post-administration. The results showed that 2 mg/kg of aficamten reduced FS%, increased left ventricular systolic dimensions, and decreased IVRT, suggesting that lower doses may be required and that further studies are necessary to establish the optimal dosage and interval [179]. A subsequent study by Sharpe et al. [180] examined dose reductions (0.3–1 mg/kg) in seven cats and found a dose-dependent reduction in left ventricular systolic function, IVRT, and LVOT pressure gradients. The authors concluded that the most appropriate dose range is 0.3–1 mg/kg, but further research is required to evaluate the long-term safety of aficamten [180]. Neither mavacamten nor aficamten is currently approved for veterinary use, with aficamten awaiting approval from the US Food and Drug Administration (FDA). However, it is hypothesised that these drugs may provide therapeutic benefits for cats with HCM [148].

Another promising therapeutic candidate for feline HCM is rapamycin, a macrolide produced by bacteria, which has completed all FDA-mandated technical phases for subclinical HCM. As of 15 January 2025, the HALT HCM pivotal trial is ongoing. Rapamycin is linked to the TOR protein, which has a mammalian homologue, mTOR [181]. The mTOR complex interacts with various proteins to form multiprotein complexes, mTORC1 and mTORC2, with differing sensitivities to rapamycin [181]. mTORC1 is responsible for positively regulating protein and lipid synthesis while negatively regulating autophagy, leading to adaptive remodelling [181]. mTORC2, in contrast, regulates glucose and lipid metabolism, stabilises cardiac physiology, and supports cardiomyocyte survival in response to elevated blood pressure [181]. Kaplan et al. [181] conducted a randomised, placebo-controlled trial with 43 cats diagnosed with subclinical HCM, administering high and low doses of rapamycin. After 180 days, the maximum ventricular wall thickness was significantly lower in cats receiving low-dose rapamycin compared to the placebo group, and the drug was well tolerated when administered orally. However, further studies are required to confirm rapamycin’s effectiveness in reducing ventricular hypertrophy [181]. Additionally, recent evidence supports the potential therapeutic role of CK-586, a cardiac myosin inhibitor. CK-586 has been shown to reduce LVOTO, increase LV systolic dimensions, and decrease FS% and ejection fraction without affecting heart rate, suggesting a promising therapeutic trend for future investigation [182].

Overall, new treatment strategies for feline HCM are essential to ensure that affected cats receive adequate therapeutic support for symptom management and disease progression. While established therapeutic protocols provide substantial benefits, cats that become refractory to treatment may require additional options. Advances in human medicine, such as myosin ATPase inhibitors (mavacamten and aficamten), have demonstrated promising results and may offer benefits to cats with HCM. Additionally, rapamycin remains in the final stages of clinical evaluation for its potential role in modulating cellular growth mechanisms. However, despite recent advancements, further studies are needed to determine the efficacy and safety of these therapies in feline populations, including their clinical outcomes, compatibility with existing treatments, and effectiveness at different disease stages.

## 9. Conclusions

Feline cardiomyopathies, particularly those characterised by a hypertrophic phenotype, can lead to severe physiological changes. The associated clinical signs range from mild to severe, significantly impacting the life expectancy of affected cats. However, research on predictive factors contributing to the development and progression of this disease remains limited. The pathophysiology of HCM primarily involves left ventricular dysfunction, characterised by concentric hypertrophy, reduced ventricular lumen, and associated haemodynamic changes. Genetic studies have identified a mutagenic component, particularly in certain feline breeds, further underscoring the need for genetic screening and early detection. Early diagnosis and disease staging are crucial for optimising therapeutic and nutritional management. Regular monitoring and routine veterinary check-ups are essential for evaluating disease progression and ensuring appropriate intervention when necessary. Although treatment approaches vary, the primary goals are to manage clinical signs, prevent disease progression, enhance quality of life, and improve overall survival. Given the complexity of this condition, further research is needed to better understand its genetic mechanisms, develop early diagnostic strategies, and explore novel therapeutic options for more effective management.

## Figures and Tables

**Table 1:** Genetic evidence for the presence of the feline hypertrophic cardiomyopathy phenotype.

Genetic Evidence	Reference
MYBPC3 A31P (Maine Coon, Munchkin)	[6,45]
MYBPC3 R820W (Ragdoll)	[7]
MYH7	[43]
ALMS1 g.92439157 G > C (Sphynx)	[44]
ALMS1 p.G3376R (Exotic Shorthair, Scottish Fold, Sphynx)	[45]
MYBPC3 A74T (Maine Coon, Bengal)	[47]
TNNT2 c.95108G > A (Maine Coon)	[49]

## Data Availability

No new data were created or analyzed in this study.

## Abbreviations

A31P	A31P mutation
ACE2	angiotensin II-converting enzyme
ACVIM	American College of Veterinary Internal Medicine
ALMS1	ALMS1 variant
AMC	arrhythmogenic cardiomyopathy
ANG II	angiotensin II
ANP	atrial natriuretic peptide
AO	aorta
ATE	arterial thromboembolism
BNP	B-type natriuretic peptide
cfDNA	cell-free DNA
CHF	chronic heart failure
citH3	citrullinated histone H3
CRI	continuous infusion
cTnI	cardiac troponin-I
DCM	dilated cardiomyopathy
ESC	European Society of Cardiology
FDA	Food and Drug Administration
HCM	hypertrophic cardiomyopathy
IL-6	interleukin 6
IV	intravenous
IVRT	isovolumetric relaxation
IVSd	interventricular septum in diastole
IVSs	interventricular septum in systole
LA	left atrium
LAA	left atrial appendage
LA/Ao	left atrium/aorta ratio
LAMP	loop-mediated isothermal amplification assay
LFD	lateral flow dipstick
LOX	lysyl oxidase
LV	left ventricle
LVFW	left ventricle free wall thickness
LVFWd	left ventricle free wall thickness in diastole
LVFWs	left ventricle free wall thickness in systole
LVOT	left ventricular outflow tract
LVOTO	left ventricular outflow tract obstruction
MAPSE	mitral annular plane systolic excursion
MCP-1	monocyte chemoattractant protein 1
mTOR	mammalian homologue TOR protein
MYBPC3	MYBPC3 gene
MYH7	MYH7 gene
NETs	neutrophil extracellular traps
NLR	neutrophil/lymphocyte ratio
NTproANP	N-terminal pro-atrial natriuretic peptide
NTproBNP	N-terminal pro-B-type natriuretic peptide
PO	orally
PVF	pulmonary venous flow
R820W	R820W mutation
RA	right atrium
RAS	renin–angiotensin system
RMC	restrictive cardiomyopathy
RV	right ventricle
RVOT	right ventricular outflow tract
SAM	systolic anterior movement
SF%	shortening fraction
SQ	subcutaneous
T4	thyroxine
TGF-β	transforming growth factor beta
TNNT2	TNNT2 intronic variant
TpTe	Tpeak–Tend
TT-LD	color-coded tissue tracking
VHS	vertebral heart size
VLAS	vertebral left atrial size
